# Parametric Cyclic Voltammetric Analysis of an Electrochemical Aptasensor for *Staphylococcus aureus* Iron-Regulated Surface Determinant Protein A Detection

**DOI:** 10.3390/mi16020162

**Published:** 2025-01-30

**Authors:** Shokoufeh Soleimani, Tracy Ann Bruce-Tagoe, Najeeb Ullah, Michael K. Danquah

**Affiliations:** 1 Department of Mechanical, Aerospace, and Biomedical Engineering, University of Tennessee, Knoxville, TN 37996, USA; rfs655@vols.utk.edu; 2 Department of Chemical and Biomolecular Engineering, University of Tennessee, Knoxville, TN 37996, USA; pvd756@vols.utk.edu (T.A.B.-T.); lzn122@utk.edu (N.U.)

**Keywords:** foodborne pathogen, *Staphylococcus aureus*, IsdA, electrochemical, aptasensor

## Abstract

Rapid and reliable detection of pathogens requires precise and optimized analytical techniques to address challenges in food safety and public health. This study focuses on the parametric characterization of an electrochemical aptasensor for *Staphylococcus aureus* (*S. aureus*) iron-regulated surface determinant protein A (IsdA) using cyclic voltammetry (CV) analysis, which offers a robust method for evaluating electrode modifications and electrochemical responses. Key parameters were optimized to ensure maximum sensitivity, including an aptamer concentration of 5 μM, an incubation time of 4 h, a potential range from −0.1 to 0.9 V, and a scan rate of 0.05 V/s. The aptasensor achieved stability and peak performance at pH 7.5 and 25 °C. These conditions were critical for detecting the IsdA protein as a biomarker of *S. aureus*. The aptasensor applicability was demonstrated by successfully detecting *S. aureus* in food samples such as milk and apple juice with high specificity and reliability. Zeta potential measurements confirmed the layer-by-layer charge dynamics of the AuNPs-aptamer-IsdA system. This work emphasizes the importance of CV in understanding the performance of the electrochemical sensor, and supports the aptasensor as a practical, sensitive, and portable tool for addressing critical gaps in foodborne pathogen detection.

## 1. Introduction

*S. aureus* is a Gram-positive bacterium and a leading cause of food poisoning, skin infections, and more severe conditions such as pneumonia and septicemia [1,2]. The challenges it presents in food safety are particularly concerning, with its contamination leading to food spoilage and severe health risks for consumers. Given the challenges posed by *S. aureus*, including the public health threat of foodborne illnesses, there is an urgent need for rapid, sensitive, and specific methods for its detection. The development of novel monitoring tools capable of accurately identifying *S. aureus*, particularly at low counts, is crucial for timely intervention, infection control, and prevention efforts.

Traditional approaches for identifying *S. aureus*, such as culture and plate counting techniques, polymerase chain reaction (PCR) assays, and enzyme-linked immunosorbent assays (ELISA) [3,4,5], have provided the backbone for diverse detection technologies. These conventional approaches have various challenges, including the need for specialized and expensive laboratory facilities, prolonged processing times, and the requirement for highly trained personnel. In response to these limitations, electrochemical detection methods have gained attention for their sensitivity, specificity, and potential for miniaturization [6,7,8]. This technique is based on electrochemistry, which focuses on the interplay between electricity and chemical reactions in relation to how electrical charges can induce or result from chemical changes. Techniques such as CV, which applies varying electrical potentials to study redox reactions and electron transfer rates, can be used to measure electrochemical responses [9,10]. Other electrochemical analysis techniques, such as electrochemical impedance spectroscopy (EIS), measure the resistance of a system to electrical flow, providing detailed information on surface properties and biofilm dynamics [11,12,13]. Electrochemical biosensing can offer a promising platform for rapid detection of *S. aureus*, combining the high sensitivity of electrochemical detection and specificity of biological recognition elements [14]. Electrochemical sensors based on aptamers (high-affinity single-stranded DNA or RNA recognition elements) have the capability to detect targets with high specificity. Aptamers have several advantages over commonly used antibodies in many aspects: easy synthesis, outstanding chemical stability, reusability, and much lower production cost. These properties set them as ideal candidates in biosensing applications, particularly for detecting pathogens. The application of aptamers in life sciences is more than just pathogen detection [15,16,17]. Aptamers can support other applications, including diagnostics, therapeutics, bioseparation, and molecular purification in the food and pharmaceutical industries. Applications range from the detection of toxins, bacteria, and viruses in food products [18,19] and the purification of biopharmaceutical products [20,21], to drug delivery systems for targeted therapies [22,23]. The ability to tailor aptamers to specific targets further extends their utility across different fields, making them an essential tool in the advancement of biosensing technologies [24,25].

The emerging demand for portable, user-friendly, and real-time pathogen detection devices in the food industry has led to significant interest in developing compact sensors capable of delivering rapid results. Screen-printed carbon electrode (SPCE) systems integrated with various nanomaterials, such as gold (Au) and silver (Ag) nanomaterials, are a common biosensing platform that can increase the sensitivity and selectivity of detection methods via enhancement of the sensing surface. For example, gold nanoparticles (AuNPs) can enhance the electrical conductivity and surface area of the electrode-sensing surface and facilitate the immobilization of bioaffinity probes such as aptamers, thereby improving the performance of the aptasensor [26,27,28,29,30].

Although AuNP-based electrochemical aptasensing demonstrates significant potential, several critical parameters remain insufficiently optimized for pathogen detection. Reich et al. (2017) utilized an electrochemical aptasensor for *S. aureus* detection but did not optimize the scan rate that is necessary to tailor the electrochemical sensitivity to the molecular context, resulting in suboptimal sensitivity [31]. Jia et al. (2014) emphasized the importance of a sensitive, specific and label-free impedimetric-based aptasensor for *S. aureus* detection yet did not provide specific electrochemical optimizations for pathogen detection [32]. These studies highlight the need for further refinement of critical electrochemical parameters, such as scan rate, potential range, and analyte concentration, to the biomolecular environment of the binding process. This study addresses these gaps by optimizing these parameters to improve sensitivity and selectivity in *S. aureus* detection.

**Scheme 1:**
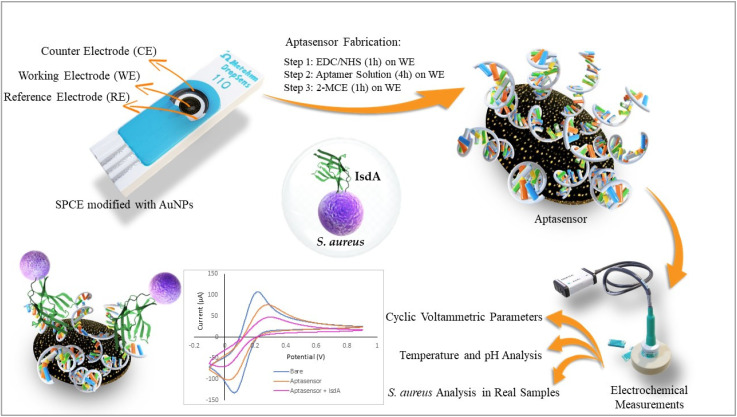
Overview of the aptasensor fabrication process for IsdA and *S. aureus* detection.

Our work focuses on the development and parametric characterization of an electrochemical aptasensor to detect *S. aureus* IsdA. The aptasensor utilizes SPCE modified with AuNPs to enhance the surface area and conductivity for aptamer immobilization. AuNPs play a vital role in improving the sensing performance by providing a high surface-to-volume ratio, facilitating aptamer binding, and enhancing electron transfer kinetics [33,34]. The combination of AuNPs and SPCE is intended to achieve high sensitivity and specificity, making it suitable for detecting *S. aureus*. To address existing gaps in the field, we investigate the optimization of critical parameters such as pH, scan rate, potential range, and aptamer concentration to maximize the performance of the aptasensor. CV was employed as the main analytical tool to study these parameters, ensuring optimal signal output and target binding efficiency. Previous studies have demonstrated the potential of aptasensors for pathogen detection, but many lack detailed parametric studies to create optimized target detection systems [15,35,36,37]. This work builds upon existing research by incorporating CV parametric analyses to optimize performance and validate the aptasensor for S. aureus detection in real food substances (Scheme 1).

## 2. Materials and Methods

### 2.1. Experimental Approach

SPCEs modified with AuNPs (SPCE/AuNPs), catalog number DRP-110GNP-U50, were acquired from Metrohm, Lombard, IL, USA. The amine coupling kit (N-Hydroxysuccinimide (NHS), and 1-Ethyl-3-(3-dimethylaminopropyl) carbodiimide Hydrochloride (EDC)), catalog number BR100050, was sourced from Cytiva, Marlborough, MA, USA. Phosphate-buffered saline 10× (PBS), catalog number AM9625, was purchased from Fisher, Waltham, MA, USA. Potassium Ferricyanide (III), K_3_[Fe(CN)₆], catalog number 702587, Potassium Ferrocyanide (K_4_[Fe(CN)_6_]), catalog number 60279, and 2-Mercaptoethanol (2-MCE), catalog number M6250, were purchased from MilliporeSigma, Burlington, MA, USA. IsdA with the amino acid sequence reported by the manufacturer as: ATEA TNATNNQSTQ VSQATSQPIN FQVQKDGSSE KSHMDDYMQH PGKVIKQNNK YYFQTVLNNA SFWKEYKFYN ANNQELATTV VNDNKKADTR TINVAVEPGY KSLTTKVHIV VPQINYNHRY TTHLEFEKAI PTLADAAKPN NVKPVQPKPA QPKTPTEQTK PVQPKVEKVK PTVTTTSKVE DNHSTKVVST DTTKDQTKTQ TAHTVKTAQT AQEQNKVQTP VKDVATAKSE SNNQAVSDNK SQQTNKVTKH NETPKQASKA KELPKT, catalog number CSB-EP763937SKW, was sourced from CUSABIO, Houston, TX, USA. IsdA-specific aptamer sequence 

(5′-gcg cac gcg ugu gua gua cac acg auc gcg cgc aca auau-3′), designed by our research group [38], was synthesized by Integrated DNA Technologies, Coralville, IA, USA. *S. aureus* (ATCC 25923), catalog number 23-021104, was purchased from Fisher Scientific, Waltham, MA, USA. Culture media, including Tryptic Soy Broth (TSB), catalog number 211768 and Tryptic Soy Agar (TSA), catalog number 236920, were purchased from Becton, Dickinson and Company, Franklin Lakes, NJ, USA, and μStat-i 400s potentiostat/galvanostat, catalog number STAT-I-400S, was purchased from Metrohm, Lombard, IL, USA.

### 2.2. Preparation of Bacterial Strains

The *S. aureus* cultures for detection analysis were prepared in our research lab at the University of Tennessee at Knoxville (UTK). A strain of *S. aureus* (ATCC strain 25923) was maintained in our research lab using TSA slants and TSB. Additionally, 0.85% sterile saline and TSB for serial dilutions of *S. aureus* were utilized. To cultivate and plate the *S. aureus* cultures for initial enumeration, TSA was employed. This process established a calibration curve correlating optical density at 600 nm (OD600) with Colony Forming Units per milliliter (CFU/mL). TSA and TSB were prepared and autoclaved at 121 °C for 20 min before being poured into sterile Petri dishes or used in detection analysis at 15 psi [39]. TSA slants were created by transferring aliquots of the prepared TSA to test tubes before autoclaving (Primus Sterilizer Co LLC, Sarpy County, Nebraska, USA). The TSA plates were then stacked and allowed to dry for 72 h at room temperature prior to use. For the dilutions, 0.85% sterile saline was prepared by dissolving 8.5 g of NaCl in 1 L of deionized water (DIW) and then sterilized using an autoclave.

### 2.3. Microscopic Characterization

Fluorescent microscopy utilizes specific dyes to illuminate target molecules, enabling their visualization on the surface of a sensor [40,41]. This technique was applied to observe the interaction between the aptamer and IsdA to assess the functionality of the aptasensor based on the distribution and binding activity of the aptamer. This visualization is critical for confirming the efficacy of the aptasensor in detecting the target IsdA molecule. We visualize the distribution and binding efficacy of the aptamer on the electrode surfaces using fluorescent imaging. This technique is essential to provide insights into the surface coverage and homogeneity of the aptamer, with fluorescence intensity variations reflecting differences in aptamer concentration and binding uniformity. It shows how the aptamer ligand density on the electrode and its IsdA binding characteristics in the conjugated state influence the surface properties of the aptasensor and help optimize the detection performance. This combined approach provides essential data for fine-tuning the aptasensor’s interface to achieve enhanced detection. Fluorescent imaging was conducted using the SP8 White Light Laser Confocal System (Leica Microsystems, Wetzlar, Germany) with an appropriate filter setup. The analysis focused on SPCE/AuNPs, the aptamer, and the interaction between the aptamer and IsdA on the electrode surface. The analysis was carried out by applying 20 μL of 6-carboxyfluorescein (FAM) dye to the surface of the aptasensor and allowing it to incubate at room temperature. Subsequently, a mixture of 20 μL FAM and 20 μL of 2 μM IsdA was added to the aptasensor’s surface and also maintained at room temperature. To prevent any photo-induced reactions, the samples were shielded with foil inside a Petri dish.

We performed scanning electron microscopy (SEM) using a Zeiss Dual Beam FIB/SEM (Oberkochen, Germany) to characterize the surface morphology of the SPCE/AuNPs. The SEM analysis of the bare SPCE/AuNPs revealed insights into the distribution and average size of AuNPs as the electrode surface to understand the conductive and binding properties of the platform. Following electrode modification with the aptamer (5 μM), SEM imaging of the aptasensor surface was conducted to confirm the successful attachment of the aptamer molecules to the AuNPs, indicative of effective functionalization for target binding. In addition, SEM analysis of the aptasensor after binding with IsdA (5 μM) was performed to examine any morphological changes upon protein–aptamer complex formation, thereby providing insights into the structural changes associated with target capture on the sensor surface. These SEM observations are essential for correlating surface characteristics with the sensor’s electrochemical performance in detecting IsdA.

### 2.4. Zeta Potential

Zeta potential measures the electric charge at the surface of a particle in a liquid, helping us understand the charge and stability of particles in suspension [42,43]. In this study, we conducted zeta potential measurements to evaluate the IsdA and IsdA-aptamer complex under varying pH conditions. Each sample, prepared at a concentration of 5 μM for both IsdA and aptamer, was analyzed in 1 mL cuvettes using water as the solvent. For the zeta potential analysis, the refractive index of water was set at 1.33, viscosity at 0.89 mPa.s, and relative permittivity at 78.36. The measurements were performed at room temperature with an equilibration time of 1 s. Automatic adjustment mode was applied for power settings with a maximum voltage of 10 V, and data quality control was set to automatic with a maximum of 1000 runs. The Smoluchowski approximation and a Henry factor of 1.5 were applied to ensure accurate results. Each sample was analyzed at three pH levels (4.5, 7.5, and 10.5) to investigate the impact of pH on zeta potential and, consequently, the binding affinity and stability of the IsdA-aptamer complex.

### 2.5. Aptasensor Fabrication

The SPCE/AuNPs electrode was activated using a 20 mL solution containing 0.4 M EDC and 0.1 M NHS (1:2 *v*/*v*) for 1 h, followed by rinsing with DIW [44]. EDC is responsible for activating carboxyl groups on the AuNPs surface by forming a reactive O-acylisourea intermediate. However, the O-acylisourea intermediate is prone to hydrolysis in aqueous conditions, which can revert to carboxyl groups and produce urea as a by-product. To improve the efficiency of the coupling reaction, NHS was added to convert the EDC-activated carboxyl groups into a more stable NHS-ester intermediate. This intermediate is less susceptible to hydrolysis and more reactive towards primary amines [45,46]. The NHS-ester-modified carboxyl groups on the AuNPs can react with primary amine groups on the aptamer, forming stable amide bonds and releasing NHS, effectively conjugating it to the AuNPs’ surface. To immobilize the aptamer onto the AuNPs-modified electrode, we applied a 5 μM solution of the aptamer to the SPCE/AuNPs electrode and left it at room temperature for 4 h. Afterward, the electrode was rinsed with PBS and DDW to remove any unbound aptamer. To block any remaining active sites on the modified electrode surface, 20 μL of 1 mM 2-MCE solution was applied and incubated for 1 h at room temperature. The electrode was then washed with PBS and DDW and dried at room temperature. The resulting aptasensor was stored in a Petri dish on the bench at room temperature for future CV and EIS analysis.

### 2.6. Aptamer Concentration and Incubation Time Effect

The concentration of aptamer and the incubation time are critical parameters in aptasensor fabrication as they directly affect the density of aptamers on the electrode surface, influencing binding efficiency and specificity. Optimizing these factors ensures sufficient target binding sites, minimizes non-specific interactions, and enhances the sensitivity of the aptasensor [47]. Furthermore, reducing the incubation time shortens the overall sensor fabrication process, which makes it more practical for rapid applications [24]. Using CV analysis, we assessed how varying aptamer concentrations and incubation times affect the electrochemical performance of the aptasensor. This was carried out by incubating the electrode with a 5 μM aptamer solution over a series of time intervals from 1 to 8 h at room temperature. We also tested how the aptasensor responds to different aptamer concentrations, using concentrations ranging from 1 to 13 μM, with each concentration incubated for 4 h. After each incubation, we measured changes in peak current across a set potential range using CV.

### 2.7. Parametric CV Analysis

In the electrochemical analysis, we first conducted CV scan rate analysis to optimize the detection performance of the aptasensor in the probe solution at pH 7.5. The scan rates were carefully selected to encompass a broad spectrum of conditions, allowing us to accurately measure the electrochemical behavior of the bare SPCE/AuNPs electrode. The rates were tested in ascending order (0.012 V/s, 0.025 V/s, 0.05 V/s, 0.1 V/s, and 0.15 V/s). This Analysis will provide a good understanding of the kinetics of electron transfer processes at each rate. For the potential range analysis, the operational windows were selected to cover a wide operational range. The potential ranges span from −0.1 to 0.9 V, −0.4 to 0.9 V, −0.8 to 0.9 V, and −0.1 to 1.0 V for the aptasensor and IsdA binding in probe solution at a pH of 7.5, to allow for a comparative evaluation of the electrochemical profiles and their implications on the performance of the aptasensor to be carried out. The potential range analysis is important to investigate and determine the optimal voltage conditions for target detection without causing any undesirable reactions or damage to the electrode surface and ensuring accurate characterization. All the CV tests were performed in the presence of the probe solution, consisting of 5 mM [Fe(CN)_6_]^−3/−4^ and 0.1 M KCl.

### 2.8. Analysis of Aptamer-Target Binding Conditions

The aptamer–target binding analysis was performed under varying pH and temperature conditions of the interaction between the aptasensor and IsdA. The effect of temperature was studied under different temperatures of IsdA: 4 °C (refrigerator), 25 °C (room temperature), and 37 °C (human body temperature), reflecting the common temperature conditions for the storage of foods and drinks and making the analyses relevant to the detection of the pathogen in food samples. The analyses were performed in the presence of 5 mM [Fe(CN)_6_]^−3/−4^ and 0.1 M KCl. We also conducted experiments to study the binding behavior of the aptasensor under different pH conditions ranging from 4.5, 7.5, and 10.5. These tests are essential to determine the conditions that enhance or inhibit the aptamer–target binding interaction, providing insights into the operational parameters and ranges vital to optimizing the performance of the aptasensor.

### 2.9. S. aureus Detection

For the bacterial detection experiments, the initial phase involved using the bacteria suspended in DIW at a concentration of 1 CFU/mL. The choice of DDW as the initial medium provides a controlled environment free of interfering substances or inhibitors. This is important to establish a baseline detection capability of the aptasensor, ensuring that the aptasensor’s response to the presence of the pathogen is due solely to the bacteria without any confounding factors. Following the initial tests in DDW, the experiments were extended to more complex matrices, specifically milk and apple juice. The rationale behind selecting these food media lies in their relevance to real-world applications, where bacterial contamination in food and beverages is a significant concern. Milk and apple juice represent common food products with distinct compositions. Milk is rich in proteins, fats, and other organic compounds [38,39], and apple juice contains sugars and acids [48,49,50]. The compositions of these media can potentially interfere with the performance of the aptasensor by affecting the binding affinity or signal response. Therefore, testing the aptasensor in these matrices is crucial to evaluate its robustness, sensitivity, and specificity in detecting bacteria in environments that closely mimic actual contamination scenarios.

## 3. Results and Discussions

### 3.1. Characterization Techniques

**Figure 1:**
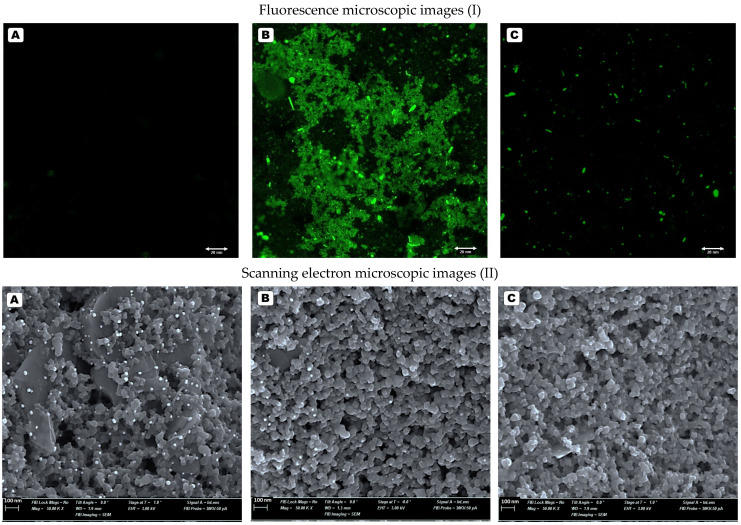
Fluorescence (**I**) and SEM (**II**) images of (**A**) bare SPCE/AuNPs, (**B**) aptasensor, and (**C**) aptasensor in the presence of 5 μM IsdA. Fluorescence images were conducted by applying 20 μL of 6-carboxyfluorescein (FAM) dye to the surface of the aptasensor.

Figure 1I shows fluorescent imaging results of the aptamer immobilization process and the subsequent binding of IsdA to the aptasensor surface. Figure 1I(A) shows the bare electrode, indicating the absence of any fluorescent signal, verifying that there are no fluorophore-labeled components present. This demonstrates a clean electrode surface devoid of non-specific interactions, setting a baseline for the subsequent experimental steps. Figure 1I(B) shows the SPCE/AuNPs surface after aptamer immobilization; a strong and uniform fluorescence signal is evident. This confirms the successful aptamers’ binding to the electrode surface. The fluorescence pattern indicates effective and homogeneous coverage. Figure 1I(C) shows the aptasensor after IsdA introduction, displaying a significant reduction in fluorescence compared to Figure 1I(B). This decrease in fluorescence intensity indicates the successful binding of IsdA to the immobilized aptamers, leading to a quenching effect as IsdA absorbs energy from the fluorophore, further confirming the interaction between the aptamer and protein. At the molecular level, the decrease in fluorescence after the binding of IsdA to the aptamer can be explained by specific energy transfer processes that dampen the fluorescent signal of the molecule attached to the aptamer. Based on Förster resonance energy transfer (FRET), when the fluorophore and IsdA are close in proximity, energy is transferred directly from the excited fluorophore to the protein rather than being released as light. This transfer of energy causes the fluorescence intensity to drop. The specific binding interaction between the aptamer and IsdA brings them within the critical distance necessary for FRET to occur, making this proximity-dependent quenching effect significant. Furthermore, static quenching may also contribute, wherein the formation of a stable non-fluorescent complex between the fluorophore and IsdA inhibits the emission of fluorescence. This quenching can be enhanced by the presence of quenching groups within IsdA, such as tryptophan residues [51,52], which further absorb energy from the fluorophore [53]. These molecular interactions are well-documented in biosensing applications, where the specific binding of a protein to an aptamer induces quenching effects, thereby confirming successful binding and interaction [38,40,41,50,54]. The presence of residual free dye indicates that some unbound fluorophore remains on the unrinsed surface. These findings collectively affirm the successful fabrication of the aptasensor and effective binding of the target protein, underscoring the potential of the aptasensor for reliable detection applications.

Figure 1II shows the SEM images detailing the sensor surface morphology during modification. Figure 1II(A) shows a densely packed surface of SPCE/AuNPs with uniformly distributed AuNPs (~25 nm in diameter). AuNPs enhance the electrode’s surface area and conductivity, creating an optimal platform for aptamer immobilization. The even distribution of AuNPs across the surface facilitates efficient electron transfer by establishing conductive pathways for the aptasensor’s performance. The morphology of the SPCE/AuNPs surface changed noticeably after aptamer immobilization, as shown in Figure 1II(B). The aptamer layer has covered the AuNPs on the electrode surface, suggesting successful immobilization. This layer introduced a resistive barrier to electron flow, impacting the redox reactions on the electrode surface. The presence of the aptamer layer confirms the successful functionalization of the electrode surface for interaction with IsdA and *S. aureus*. Figure 1II(C) shows the changes in morphology after the introduction of 5 μM IsdA on the aptasensor surface. IsdA binding caused a rearrangement or aggregation of surface structures, potentially increasing the surface roughness. The observed morphological change supports the effective capture of IsdA by the aptamer [55,56].

### 3.2. Electrochemical Analysis of Aptasensor Binding

**Figure 2:**
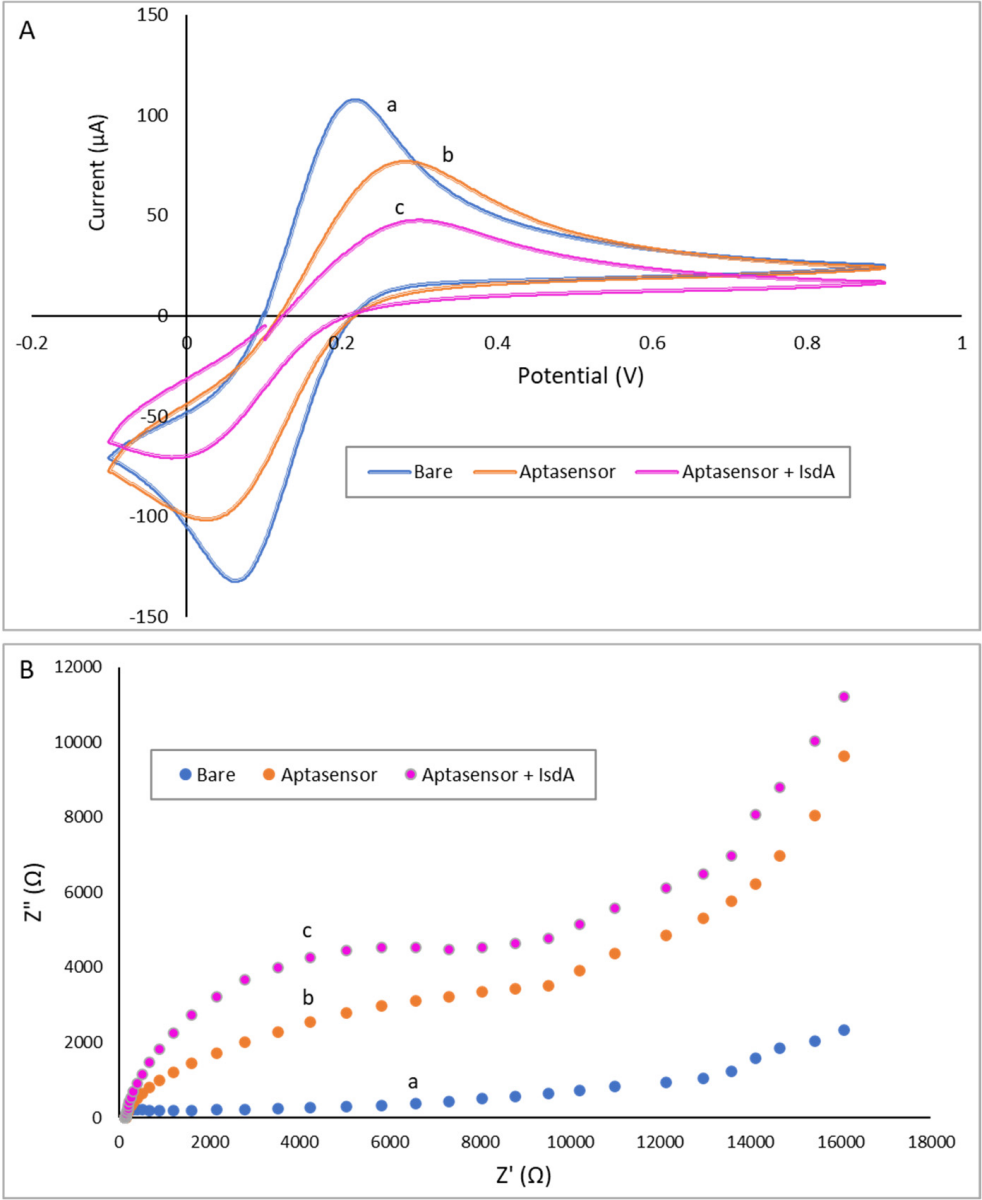
(**A**) CVs and (**B**) EISs of the different electrode states in the presence of 5 mM [Fe(CN)_6_]^−3/−4^ and 0.1 M KCl probe solution at a pH of 7.5, where curve (a) is related to bare SPCE/AuNPs, curve (b) is related to the aptasensor, and curve (c) is for the aptasensor in the presence of IsdA (5 μM).

The CV and EIS results shown in Figure 2A,B indicate changes in the electrochemical responses as the electrode undergoes successive modifications. Curve (a) represents the bare electrode, curve (b) corresponds to the aptasensor after aptamer immobilization, and curve (c) represents the aptasensor after IsdA binding. The CV and EIS responses of the bare electrode serve as the baseline for analysis. The CV data shows the highest peak current, indicating the free transfer of electrons across the electrode surface. This observation aligns with the EIS results, which indicate minimal charge transfer resistance (Rct), confirming the absence of molecular modifications impeding electron transfer. After immobilizing the aptamer on the SPCE, a reduction in peak current was observed, accompanied by an increase in the Rct value in the EIS spectrum. This decrease is primarily attributed to the aptamer layer, which imposes both steric and electrostatic barriers to electron transfer. The steric effect of aptamers plays a critical role in creating resistive barriers to electron flow when they are immobilized on the electrode surface, corresponding to the 3D structures of the aptamer [47,57]. Once immobilized, these structures can physically obstruct the electrode surface, preventing redox-active species from accessing the conductive surface. This obstruction on the electrode surface reduces the available area for electron transfer, leading to decreased peak currents in the CV profile. In addition, the large molecular size of aptamers contributes to this steric hindrance, creating a dense layer that limits the diffusion of electroactive species toward the electrode, further impairing electron transfer efficiency [58]. Furthermore, the electrostatic repulsion between the negatively charged backbone of the immobilized aptamers and negatively charged analytes in the probe solution increases this resistive effect [59]. The combined steric and electrostatic hindrances lower the conductivity and increase the resistance of the system. Aptamers, by their structured configuration and negative charge, restrict the accessibility of electroactive species to the electrode surface. This is quantitatively explained by the Butler–Volmer equation [60]:

(1) $$i=i_{0}(e^{-\frac{\alpha nF}{RT}\left(E-E_{0}\right)}+e^{\frac{\left(1-\alpha \right)nF}{RT}\left(E-E_{0}\right)})$$

where i represents the current, i_0_ is the exchange current density, α is the transfer coefficient, n is the number of electrons transferred, F is the Faraday constant, R is the universal gas constant, T is temperature, E is the electrode potential, and E_0_ is the standard electrode potential. The introduction of the aptamer layer to the electrode surface results in an increased resistance, which reduces i_0_ and effectively decreases the overall electron transfer rate. Further modification with IsdA leads to an additional decrease in peak current in the CV profile and a corresponding increase in Rct in the EIS spectrum. The IsdA binding enhances both the steric and electrostatic barriers, compounding the restriction of electron flow. The EIS data emphasizes the compounded resistance by displaying a larger semicircular diameter in the Nyquist plot, reflecting the cumulative effects of the aptamer and IsdA layers on the electrode surface. The stepwise reduction in peak currents and the progressive increase in Rct from the bare electrode to the aptasensor with IsdA binding explains the intrinsic balances between specificity and sensitivity in the aptasensor design. While the modifications enhance the ability of the aptasensor to recognize and bind to the target analyte specifically, they can impose significant challenges to the electron transfer processes essential for signal generation in electrochemical sensing. The trade-off between optimizing signal strength and recognition of the analyte in aptasensor design centers around balancing sensitivity with specificity. Increasing the surface coverage of aptamers can enhance the recognition capability due to the more significant number of binding sites for the target analyte. However, this can introduce steric hindrances and electrostatic repulsion, which in turn reduce electron transfer and thus decrease signal strength [9]. Optimizing the number of immobilized aptamers is critical to ensure that the signal-to-noise ratio remains high while maintaining strong analyte recognition. The use of nanomaterials, such as AuNPs, can mitigate some of the signal loss by enhancing conductivity [61]. However, higher concentrations of aptamer immobilization can lead to crowding and inefficient signal generation [62]. This balance is essential in designing aptasensors that maximize both sensitivity and selectivity, ensuring accurate detection without compromising the signal [63,64]. Our previous work provided a foundation to build an understanding of the design of the aptasensor, for improved sensitivity and specificity [44].

### 3.3. Zeta Potential Analysis

The zeta potential measurements (Table 1) for IsdA and its complex with the aptamer provide insights into their surface charge properties across different pH levels. For IsdA alone, the mean zeta potential at pH 4.5 is −3.25 mV, which becomes more negative at a pH of 7.5 (−7.01 mV) and slightly less negative at a pH of 10.5 (−5.58 mV). This trend suggests that at a neutral pH, IsdA reaches its peak negative charge, likely due to the deprotonation of acidic amino acid residues. The less negative zeta potential at pH 4.5 may be due to protonation, which reduces the protein’s net negative charge, while the slight decrease in negativity at pH 10.5 could result from partial denaturation or structural changes affecting surface charge distribution. In the IsdA-aptamer complex, the mean zeta potential shows different patterns. At pH 4.5, the complex has a zeta potential of −3.61 mV, similar to that of IsdA alone, indicating limited additional electrostatic interaction at acidic pH. However, at pH 7.5, the zeta potential is significantly less negative (−2.42 mV). This suggests that the binding at pH 7.5 leads to some charge neutralization or surface shielding, potentially from optimal aptamer–protein interaction. At pH 10.5, the zeta potential becomes more negative again (−5.90 mV), possibly due to partial destabilization of the complex in basic conditions, which could expose more negative surface regions or disrupt binding. This analysis indicates that pH 7.5 provides a viable environment for IsdA-aptamer interaction, providing effective binding and minimal repulsion [65,66,67].

### 3.4. Analysis of Aptamer Concentration and Incubation Time Effect

**Figure 3:**
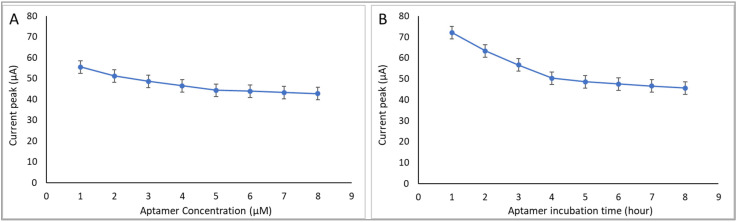
CV analysis of aptamer immobilization optimization: (**A**) indicates the influence of aptamer concentration on peak current and surface modification efficiency, and (**B**) indicates the incubation time effect on peak current stability and effective aptamer binding at the sensor surface. Experiments were conducted in the presence of 5 mM [Fe(CN)_6_]^−3/−4^ and 0.1 M KCl probe solution.

In Figure 3A, we analyzed the effect of increasing aptamer concentration on the peak current. As the aptamer concentration increases, a gradual decrease in the current peak is observed. This behavior can be attributed to the increasing surface coverage of the aptamer molecules, which leads to enhanced blocking of electron transfer at the electrode interface. Higher aptamer concentrations create a denser molecular layer, introducing steric hindrance and electrostatic repulsion, thereby impeding the movement of redox-active species toward the electrode surface. This phenomenon is well-aligned with the Butler–Volmer equation [60], which shows that increased surface resistance reduces the exchange current density, resulting in diminished electrochemical signal output. The results indicate that a concentration of 5 μM achieves optimal aptamer loading, providing a balance between effective immobilization and maintaining sufficient electron transfer. Concentrations higher than 5 μM can lead to oversaturation, causing a decrease in binding efficiency due to steric crowding and intermolecular interactions among the aptamers. Similarly, Figure 3B illustrates the impact of incubation time on the current peak. Extending the incubation time from 1 to 8 h revealed a steady decline in current, stabilizing around 4 h. This stabilization indicates that the electrode surface reaches saturation, with no further significant increase in aptamer binding efficiency beyond this period. Prolonged incubation times may also allow for non-specific interactions, which can reduce the performance of the sensor. Selecting a 4 h incubation time provides sufficient aptamer attachment without compromising the electrochemical response. This study optimizes the performance of the aptasensor, ensuring that the aptamer concentration and incubation time achieve a high density as the functional recognition elements while maintaining electron transfer capabilities.

### 3.5. CV Scan Rate Analysis

**Figure 4:**
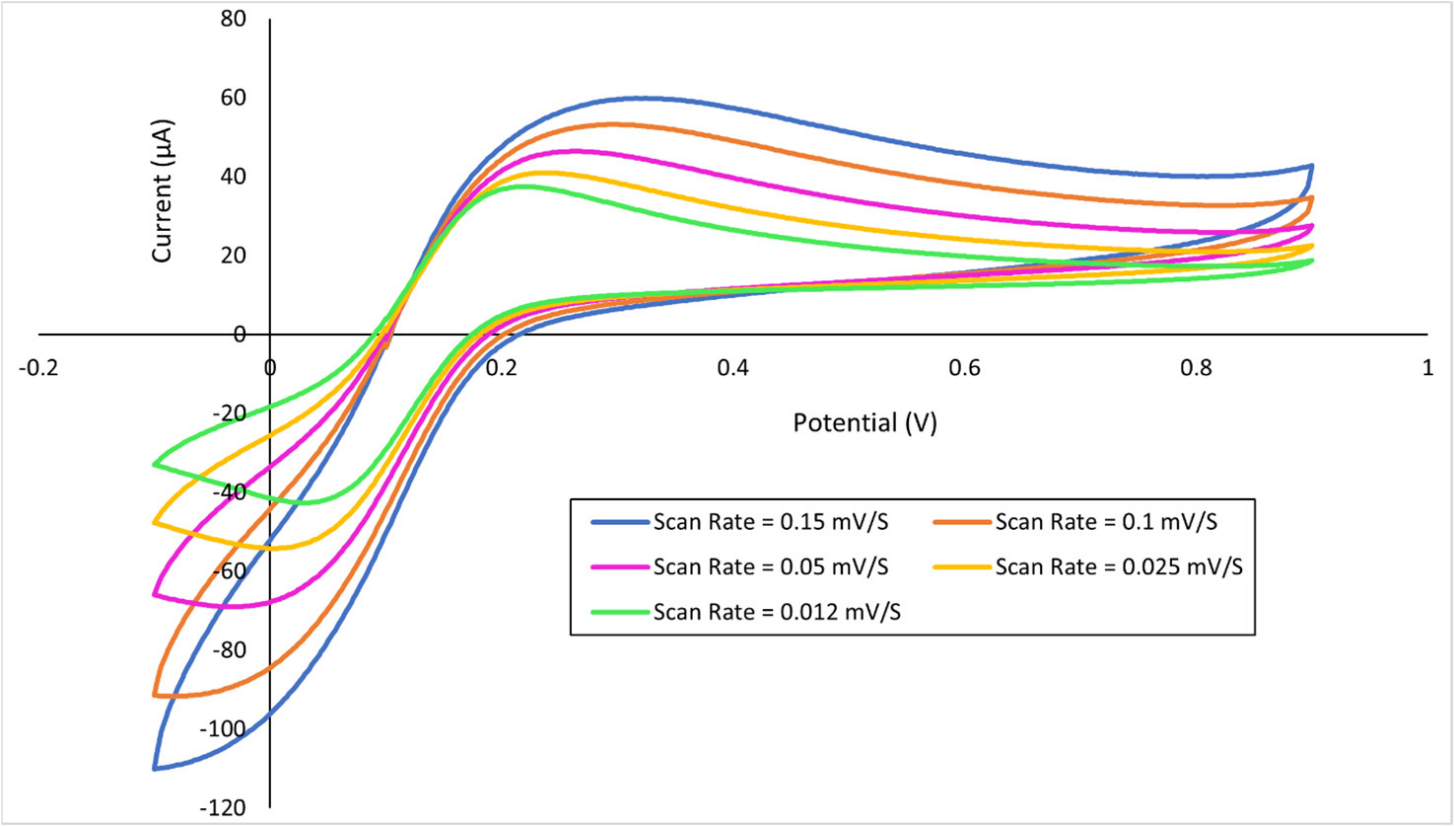
CV curves for analyzing the scan rate effect on IsdA (5 μM) detection by the aptasensor in the presence of [Fe(CN)_6_]^−3/−4^ and 0.1 M KCl at pH 7.5. Experiments were conducted at five different scan rates: 0.15 mV/s, 0.1 mV/s, 0.05 mV/s, 0.025 mV/s, and 0.012 mV/s.

The CV responses of the aptasensor for detecting IsdA at various scan rates (0.012, 0.025, 0.05, 0.1, and 0.15 mV/s) were systematically investigated as shown in Figure 4. The baseline scan rate of 0.05 mV/s was used for comparison to understand the effects of changing scan rates on the electrochemical behavior of the aptasensor. The peak current (Ip) increased with the scan rate, consistent with the Randles–Sevcik equation (Equation (2)), which describes the relationship between peak current and scan rate for diffusion-controlled processes, where Ip is the peak current, n is the number of electrons involved, A is the electrode area, D is the diffusion coefficient, C_0_ is the analyte concentration, and v is the scan rate [68]. The peak current increased with increasing scan rate from 0.012 mV/s to 0.15 mV/s, indicating that higher scan rates enhance the electron transfer kinetics, resulting in more significant current responses.

(2) $$Ip={(2.69\times 10}^{5})n^{\frac{3}{2}}{AD}^{\frac{1}{2}}{C₀v}^{\frac{1}{2}}$$

At the highest scan rate of 0.15 mV/s, the peak current is the highest, which agrees with the expected proportional relationship between the peak current and the square root of the scan rate. This increase in peak current suggests that the electron transfer process is significantly more efficient at higher scan rates. However, this can also introduce capacitive currents and distort the CV curve by contributing to non-faradaic processes, making it challenging to discern the actual redox peaks accurately. As the scan rate decreases, the peak current also decreases, as seen in the curve for the lowest scan rate of 0.012 mV/s. This lower peak current indicates slower electron transfer kinetics, as the system has more time to equilibrate, resulting in sharper but smaller peaks. The curves at 0.1 mV/s, 0.05 mV/s, and 0.025 mV/s show intermediate behaviors, where the peak currents progressively decrease with decreasing scan rates.

The peak potential (Ep) shifts slightly with changing scan rates. For reversible systems, this shift is minimal; however, for quasi-reversible or irreversible systems, significant shifts can occur due to the slower electron transfer kinetics not keeping up with the increased scan rates [69,70]. In this case, the potential shifts are indicative of the kinetic limitations of the system, where higher scan rates may result in peaks appearing at slightly more positive potentials for oxidation and more negative potentials for reduction. The scan rate also influences the shape of the CV curves. Higher scan rates, such as 0.15 mV/s, show broader peaks due to the increased capacitive currents and faster electron transfer kinetics. Conversely, lower scan rates, such as 0.012 mV/s, result in sharper peaks as the system has more time to reach equilibrium, reducing capacitive contributions and allowing more precise identification of redox processes. In addition, the scan rate affects the sensing information obtainable from the CV. Varying the scan rate provides insights into the electron transfer kinetics and whether the process is diffusion-controlled or involves adsorption. For instance, the peak current’s proportionality to the square root of the scan rate indicates diffusion control, while a direct proportionality would suggest adsorption control [71,72].

The scan rate plays a crucial role in determining the efficiency of electron transfer within the aptasensor system during IsdA detection. At higher scan rates, electron transfer kinetics are accelerated, allowing the system to capture dynamic changes in redox reactions more rapidly. However, this may lead to broader peaks and a potential shift due to kinetic limitations, as the electron transfer process may not fully equilibrate during the fast scan. Conversely, lower scan rates provide the system with more time to equilibrate, resulting in lower peaks and better resolution of redox processes, but at the cost of sensitivity [73,74]. During the detection of IsdA with the aptasensor, lower scan rates are likely more beneficial for capturing the specific binding event, as they reduce the influence of capacitive currents and non-faradaic processes, allowing for a clearer signal of IsdA-aptamer interaction.

In practical terms, the scan rate must be optimized for sensitivity and resolution. Lower scan rates enhance sensitivity and detection limits due to the sharper and well-defined peaks. In comparison, higher scan rates provide faster analysis but may compromise sensitivity and resolution due to broader peaks and increased capacitive currents. The optimal scan rate for detecting IsdA based on the study of the CV curves is the scan rate of 0.05 mV/s. At this scan rate, the peak current is sufficiently high to provide a robust and detectable signal without the distortion seen at higher scan rates, which increases capacitive currents and obscures the actual electrochemical behavior. Lower scan rates, such as 0.012 mV/s, yield sharper peaks but with significantly lower currents, reducing sensitivity and making detection more challenging. The 0.05 mV/s scan rate maintains a good balance between peak height and sharpness. It minimizes the influence of capacitive currents, ensuring the observed peaks are primarily due to faradaic processes related to IsdA detection, leading to more accurate and reliable measurements. Additionally, this scan rate provides an adequate balance between kinetic and equilibrium conditions, offering insights into electron transfer kinetics while allowing sufficient time for the system to reach a quasi-equilibrium state, which is crucial for meaningful data. It also provides a practical compromise between speed and data quality, as lower scan rates, although more detailed, take longer and are impractical for routine analysis or high-throughput screening.

In comparison to the optimal scan rate of 0.05 mV/s observed for IsdA detection, other research has reported varying optimal scan rates depending on the molecular environment, target analyte, and the specific aptasensor design. Liu et al. (2015) found that for thrombin detection using an electrochemical aptasensor, an optimal scan rate of 0.1 mV/s provided the best balance between sensitivity and peak resolution. This higher scan rate allowed for quicker analysis, though it introduced some capacitive currents, which were accounted for in the data interpretation [75]. Similarly, Wang et al. (2017) [76] identified a scan rate of 0.1 V/s as optimal for aptasensor-based detection of human breast cancer cells, demonstrating sharper peaks and reduced non-faradaic processes, though at the cost of slower data acquisition. The differences in optimal scan rates across these studies can be attributed to variations in the target molecules, the electrochemical environment, and the type of nanomaterials used in sensor fabrication. The scan rate selected in this study, 0.05 mV/s, allows for a balance between faster electron transfer kinetics and the need for accurate, reliable detection without significant distortion from capacitive effects.

### 3.6. CV Potential Range Analysis

**Figure 5:**
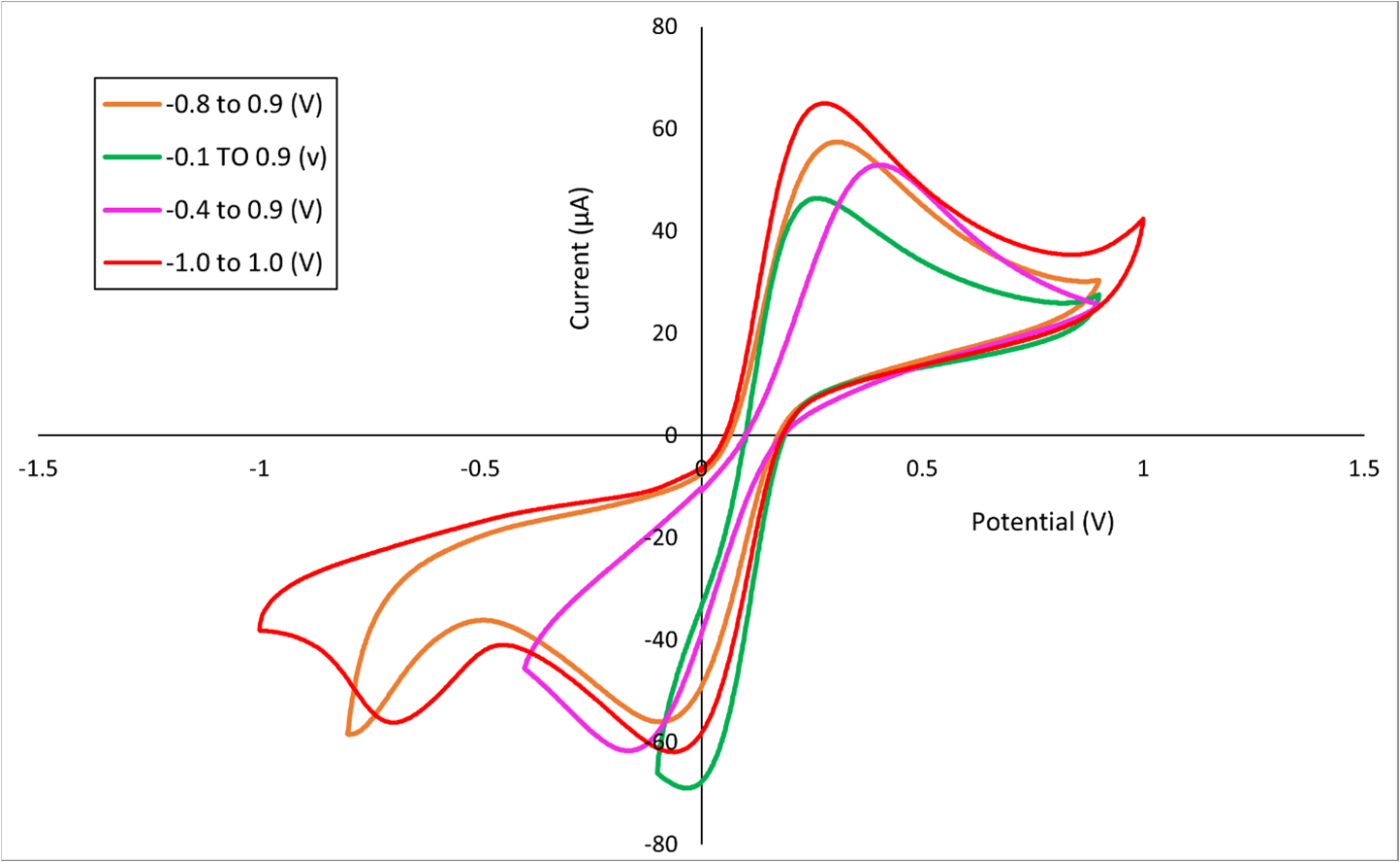
CV curves for analyzing the potential range effect on IsdA (5 μM) detection by the aptasensor in the presence of [Fe(CN)_6_]^−3/−4^ and 0.1 M KCl at pH 7.5. Experiments were conducted using 50 μL of IsdA (5μM) each time in the presence of 50 μL of probe solution at room temperature.

The analysis of CV under varying potential ranges, presented in Figure 5, shows significant changes compared to the baseline range of −0.1 to 0.9 V. The observed changes in peak current and the shape of the CV curves are due to several electrochemical factors influencing the binding condition. For the potential range of −0.4 to 0.9 V, the peak current is higher than the baseline. This increase can be attributed to the extended potential range, which allows more thorough diffusion of the electroactive species to the electrode surface. The electroactive species refers to the molecules or ions participating in the redox reactions at the electrode surface [77]. In this study, the electroactive species is IsdA, which interacts with the immobilized aptamers on the SPCE surface and the redox probe solution (5 mM [Fe(CN)_6_]^−3/−4^ and 0.1 M KCl). The ferricyanide/ferrocyanide redox couple serves as an electron mediator, facilitating electron transfer between the electrode and the electrolyte, while KCl acts as a supporting electrolyte, improving the ionic conductivity of the solution and stabilizing the redox reactions. The AuNPs enhance the conductivity and surface area of the electrode, promoting efficient electron transfer. When the aptamer binds to IsdA, the electron transfer is altered, which is reflected in the changes observed in the CV profile. The electroactive species collectively contribute to the observed shifts in current during the redox reactions at the electrode–electrolyte interface.

The additional negative potential contributes to a greater extent to the redox process, thereby increasing the peak current. However, the increase is moderate, indicating that the potential extension to −0.4 V is still within the optimal electrochemical window for the system without causing significant side reactions or capacitive current increases. In the case of the potential range of −0.8 to 0.9 V, the peak current further increases compared to the −0.4 to 0.9 V range. This suggests that the diffusion-limited region’s contribution is more pronounced, enhancing the electron transfer kinetics. However, the peak becomes broader, which can be attributed to increased capacitive currents at the more negative potential, adding to the background noise and potentially obscuring some faradaic processes. The broader peak also suggests a slight shift in the reversibility of the redox reaction, indicating slower kinetics at these extreme potentials.

The potential range of −1.0 to 1.0 V shows the highest peak current among all the tested ranges. This substantial increase can be explained by the inclusion of a more extensive diffusion-limited region and a higher driving force for the electron transfer process. However, the peak broadening is more significant, indicating increased capacitive currents and possible involvement of side reactions such as electrolyte breakdown. This could lead to additional faradaic processes that contribute to the current, resulting in the highest peak but also the broadest and potentially less defined peaks. In all cases, the shifts in peak potential with a widening potential range suggest changes in the reversibility and kinetics of the electrochemical processes. The redox reaction may become less reversible at more negative potentials, leading to slower electron transfer kinetics and broader peaks. Additionally, the increased capacitive currents at higher negative potentials contribute to the overall shape change, adding to the background current and making it difficult to distinguish the accurate faradaic processes. Therefore, the potential range of −0.1 to 0.9 V is the most suitable among the tested ranges. It provides well-defined and sharp peaks, minimizes capacitive currents and side reactions, and maintains a good balance between peak height and clarity. This range ensures reliable and accurate detection of the redox processes, making it the preferred choice for further electrochemical analysis.

### 3.7. Analysis of the Effect of Temperature

**Figure 6:**
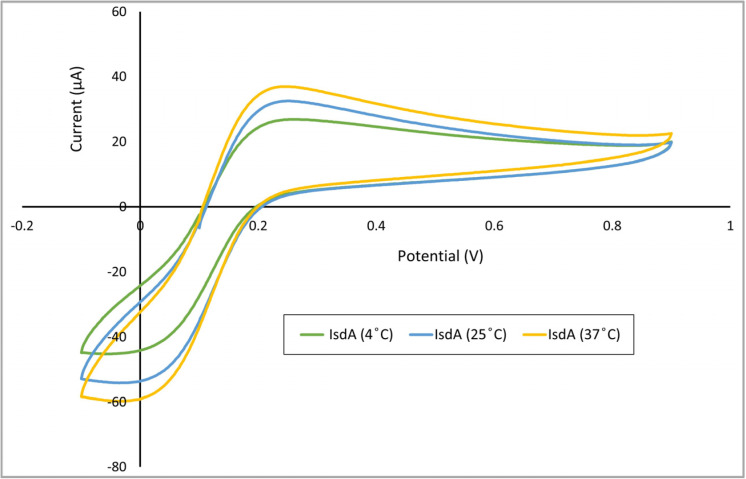
CV curves for analyzing the temperature effect on IsdA (5 μM) detection by the aptasensor in the presence of [Fe(CN)_6_]^−3/−4^ and 0.1 M KCl at pH 7.5, at three different temperatures (37 °C, 25 °C, 4 °C).

The CV responses of the electrochemical aptasensor for detecting IsdA at 4 °C, 25 °C, and 37 °C were analyzed (Figure 6) to evaluate the impact of temperature on electrochemical detection of IsdA. The CV curve at 4 °C shows the lowest peak current. This is consistent with the reduced kinetic energy and slower diffusion rates at lower temperatures [78,79], which result in less efficient electron transfer processes. Consequently, the electrochemical activity is reduced, as indicated by the lower current response. The reduced mobility of the redox-active species (ferricyanide [Fe(CN)_6_]^−3^ and ferrocyanide [Fe(CN)_6_]^−4^ ions that undergo reversible redox reactions, where [Fe(CN)_6_]^−3^ is reduced to ([Fe(CN)_6_]^−4^ and vice versa) at this temperature is the primary reason for the observed lower peak current. Increasing the temperature to 25 °C, which represents room temperature, the peak current increased. This increase can be attributed to the increased kinetic energy of the molecules, leading to higher diffusion coefficients and more efficient electron transfer processes. From Fick’s law of diffusion (Equation (3)) [80],

(3) $$J=-D\frac{\partial \;C}{\partial \;x}$$

where J represents the flux of the species (rate of transfer per unit area), which is the amount of substance that moves across a unit area per unit of time, typically expressed in units of moles per square meter per second. D is the diffusion coefficient, a measure of how easily the species can diffuse through the medium. It depends on factors such as temperature, the size of the molecule, and the viscosity of the medium. Units are typically reported in square meters per second (m^2^/s). $\frac{\partial C}{\partial x}$ is the concentration gradient of the species, representing the change in concentration (C) with respect to distance (x). It shows how concentration varies over a spatial interval, influencing the direction and magnitude of diffusion. Units are typically in moles per cubic meter per meter (mol/m^3^/m or mol/m^4^). As D increases with temperature and the peak current (Ip) in CV is proportional to the square root of D, the relationship can be described by the equation in Equation (4):

(4) $$Ip\propto \sqrt{D}$$

The increase in diffusion coefficient at higher temperatures facilitates the movement of redox species toward the electrode surface, thereby improving the overall electrochemical performance of the sensor. The peak observed at 25 °C is well-defined and sharper, reflecting an optimal balance between kinetic energy and molecular diffusion. At 37 °C, the peak current increased, demonstrating the highest current responses among the three tested temperatures. This further enhancement in current can be explained by the higher kinetic energy and diffusion coefficients at this elevated temperature, resulting in more pronounced redox activity. The elevated temperature increases the rate of electron transfer processes, enhancing the overall electrochemical response. However, it is essential to note that extreme temperatures can affect the structural integrity of IsdA, the binding efficacy of the aptamer, and the material characteristics of the electrode system, potentially affecting the performance of the aptasensor system. While the peak current is higher than at 37 °C, it is not significantly higher than at 25 °C, suggesting that room temperature analysis has the potential to provide optimal conditions for detection of IsdA using the electrochemical aptasensor.

### 3.8. Analysis of pH Effect on IsdA/Aptamer Complex Formation

**Figure 7:**
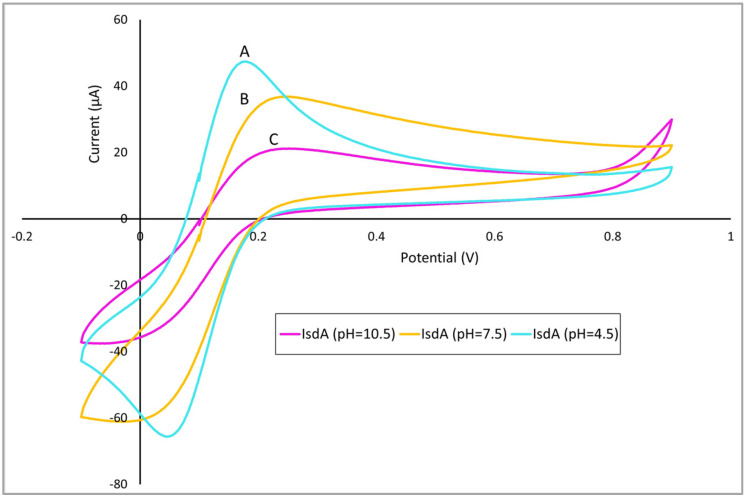
CV curves for analyzing the pH effect on IsdA (5 μM) detection by the aptasensor in the presence of [Fe(CN)_6_]^−3/−4^ and 0.1 M KCl. (A) is the IsdA-aptamer complex at pH = 4.5, (B) is the IsdA-aptamer complex at pH = 7.5, and (C) is the IsdA-aptamer complex at pH = 10.5.

The combined analysis of CV data (Figure 7) and zeta potential measurements (Table 1) reveals the impact of pH on the IsdA-aptamer’s interaction with the aptasensor. At pH 4.5, the zeta potential for aptamer-IsdA complex is relatively low (−3.61 mV), indicating minimal electrostatic repulsion. This low stability may result in weaker binding between the aptamer and IsdA, allowing more electrons to reach the electrode surface, which is reflected in the higher CV peak current observed in acidic conditions. The protonation of acidic amino acids such as aspartic acid (Asp) and glutamic acid (Glu) at low pH could reduce the overall negative charge on the protein, disrupting effective binding with the aptamer. At pH 7.5, IsdA exhibits a more negative zeta potential (−7.01 mV), suggesting a charge state conducive to binding with the aptamer. Aptamer-IsdA complex at this pH has a slightly less negative zeta potential (−2.43 mV), likely due to a favorable balance of charged residues such as lysine (Lys) and arginine (Arg), which maintain the protein’s structural integrity for aptamer binding. The lower CV peak current at pH 7.5 supports this stronger binding, as efficient electron-blocking by the complex reduces electron transfer to the electrode surface. In basic conditions (pH 10.5), the zeta potential for aptamer-IsdA complex becomes more negative (−5.90 mV), likely due to the deprotonation of amino acids such as histidine (His) and tyrosine (Tyr) in IsdA structure, which alters its surface charge and potentially disrupts binding stability. IsdA zeta potential at this pH (−5.58 mV) is also less negative compared to pH 7.5, reflecting a possible structural change from deprotonation that reduces effective binding with the aptamer. The CV data at pH 10.5, which shows a reduced peak current, aligns with weaker electron transfer blockage, confirming a less effective binding in alkaline conditions. The zeta potential data aligns with the redox shifts observed in the cyclic voltammetry, illustrating that pH-induced changes in the surface charge of IsdA-aptamer complex directly affect their electrochemical interactions. At lower pH, minimal electrostatic repulsion leads to reduced electron transfer blockage, resulting in higher peak currents. Conversely, at neutral pH, more favorable electrostatic conditions for binding reduce peak currents due to increased electron blocking, while at higher pH, destabilized binding due to increased negativity further diminishes electron transfer efficiency. This interplay between zeta potential and redox activity highlights how electrostatic changes influence the electrochemical properties of the complex [65,66,67].

### 3.9. S. aureus Detection in Real Samples

**Figure 8:**
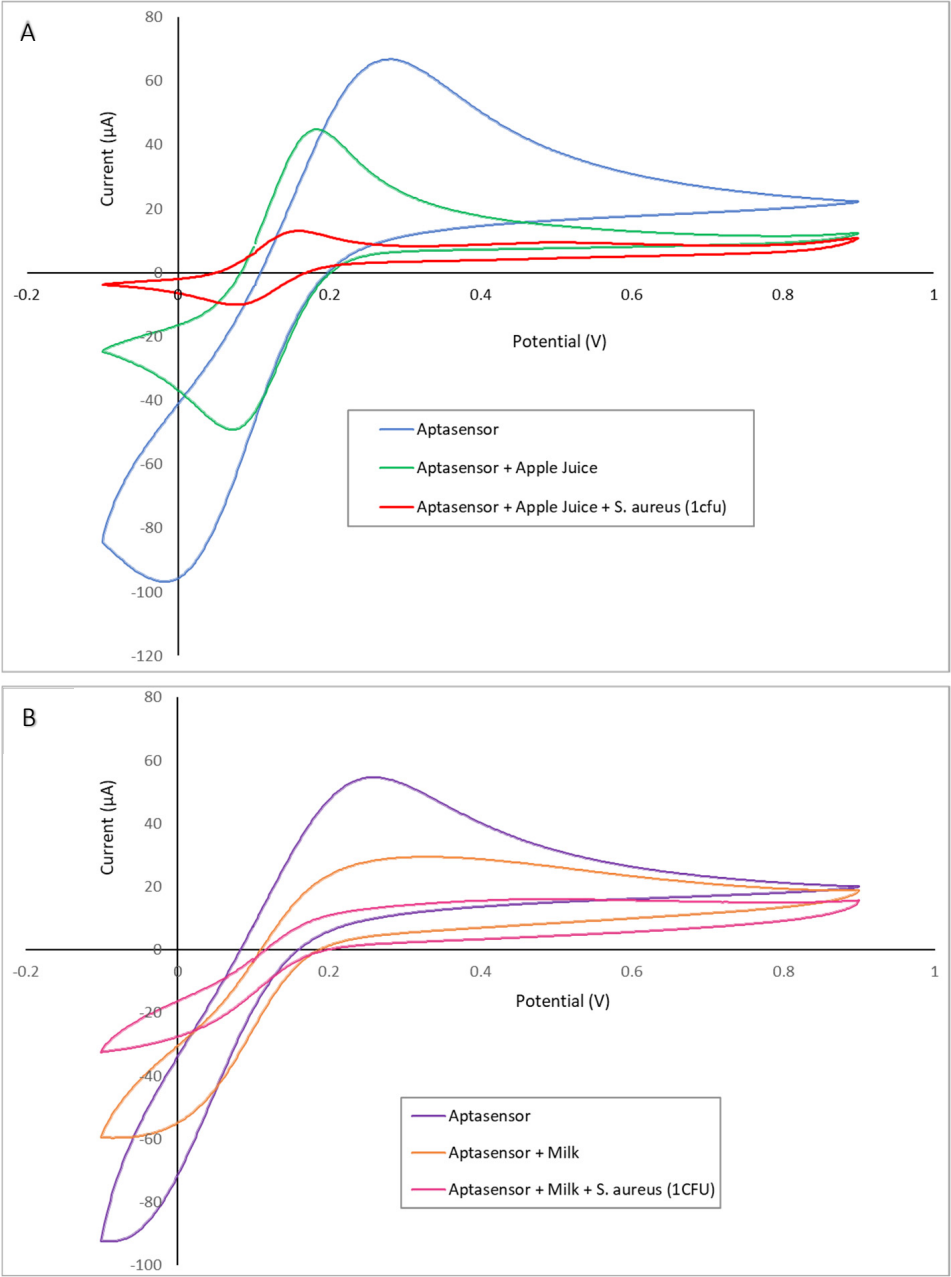
CV curves for the detection of *S. aureus* in real samples by the aptasensor in the presence of [Fe(CN)_6_]^−3/−4^ and 0.1 M KCl. (**A**) is CV for apple juice, and (**B**) is CV for milk. Experiments were conducted using 1 CFU/mL of *S. aureus* at room temperature.

We progressed the testing of the aptasensor to evaluate the presence of the pathogen in real food substances, such as apple juice and milk, as shown in Figure 8. The experiments aimed to validate the efficacy and adaptability of the aptasensor to real-world conditions where the pathogen can pose significant health risks. Figure 8A shows the CV responses of the aptasensor in apple juice, both with and without *S. aureus* contamination. The initial testing in the apple juice without bacteria shows a decreased peak current compared to the baseline, which is the aptasensor in the presence of the probe solution. This reduction is attributed to the presence of organic compounds and acids in the apple juice, which may be deposited on the electrode surface or have non-specific interactions with the aptamer, potentially hindering the electron transfer at the electrode surface. A further decrease in peak current observed upon the addition of *S. aureus* to the apple juice validates the capability of the aptasensor to detect the presence of the pathogen, as the binding of *S. aureus* to the aptasensor adds additional barriers to electron transfer, demonstrating a specific response to the biological analyte. Figure 8B presents the CV curves with a similar trend. The introduction of milk caused a reduction in the peak current. However, the effect is more pronounced than in apple juice, potentially due to the biochemical and compositional complexity of milk in terms of the presence of diverse proteins and fat molecules, forming a blocking layer on the electrode surface and impeding electron flow more substantially. The subsequent addition of *S. aureus* resulted in an even more significant decrease in the peak current. The interaction between the aptamer and the IsdA protein on the *S. aureus* surface leads to complex formation on the aptasensor electrode, which impedes the electron transfer necessary for higher current outputs. In our previous work [44], we evaluated the critical performance metrics of the aptasensor and determined its limit of detection to be 0.2 CFU. We also tested and validated the specificity and stability of the aptasensor. These findings underscore the functionality of the aptasensor in real food matrices, demonstrating its ability to detect bacterial contamination amidst the presence of interfering substances commonly found in food products. The consistent decrease in the peak current upon the addition of bacteria across different matrices confirms that the aptasensor responds specifically to the target organism, *S. aureus*. This specificity is crucial for practical applications and ensures that the aptasensor can reliably detect the presence of the pathogen in food materials, potentially providing an essential tool for food safety monitoring.

### 3.10. Limitations and Challenges

The developed electrochemical aptasensor demonstrated promising outcomes for the detection of *S. aureus* in milk and apple juice. 

However, some limitations and challenges must be addressed to fully realize its potential in practical applications. A key limitation lies in the applicability of the sensor to more complex food matrices, such as those with high fat or protein content, which may introduce significant background noise, non-specific binding, or interference with electrochemical signals. These factors can compromise sensitivity and specificity, necessitating further validation across a broader range of food products. Environmental and operational stability also pose challenges, as the optimal conditions for aptasensor performance (pH 7.5) and room temperature (25 °C) may not always align with the diverse environments encountered in real-world applications. Extreme temperatures or pH variations could disrupt the aptamer–protein interaction or compromise the structural integrity of the sensor. Similarly, while the fabrication of the aptasensor was efficient at a laboratory scale, transitioning to large-scale production presents hurdles related to consistency in electrode functionalization, AuNP deposition, and quality control, which are all critical for reproducibility and commercial viability. Although the CV parameters were optimized to enhance the signal, further advancements in signal amplification strategies, such as integrating nanocomposites or employing advanced signal processing algorithms [81], could significantly improve detection sensitivity and broaden the applicability of the aptasensor.

## 4. Conclusions

In this work, we developed and performed a parametric CV characterization of a portable *S. aureus* aptasensor. The analysis focused on the effect of scan rate and voltage range on the electrochemical signal output, as well as the impact of environmental conditions such as pH and temperature on the detection performance. Such insights are critical for fine-tuning the functional parameters of the aptasensor for a specific target, significantly enhancing the detection performance of the aptasensor. The optimal conditions for aptamer immobilization and IsdA interaction were determined, ensuring reliable and reproducible detection performance. The application of the aptasensor for *S. aureus* detection in food samples confirms its robustness and adaptability. This work shows a significant step in aptasensor application for real-time pathogen detection applications in actual food samples where rapid and accurate detection of microbial contaminants is critical. The study not only supports the continued refinement of electrochemical aptasensing methods for bacterial pathogens but also underscores the potential for their broader application in ensuring food safety and public health.

## Figures and Tables

**Table 1:** Zeta potential result for aptamer, IsdA, and Aptamer/IsdA complex.

Measurement	IsdA			Aptamer/IsdA Complex		
	pH = 4.5	pH = 7.5	pH = 10.5	pH = 4.5	pH = 7.5	pH = 10.5
Mean zeta potential (mV)	−3.25	−7.01	−5.58	−3.61	−2.42	−5.90
Standard deviation (mV)	±1.72	±1.74	±1.48	±1.20	±2.32	±1.48
Electrophoretic mobility (μm·cm/Vs)	−0.25	−0.54	−0.43	−0.28	−0.18	−0.46
Conductivity (mS/cm)	19.09	16.95	18.22	16.92	23.28	17.36
Distribution peak (mV)	−1.74	3.24	−2.16	−3.71	−0.65	0

## Data Availability

Data is available upon request.
